# The debatable presence of PIWI‐interacting RNAs in invasive breast cancer

**DOI:** 10.1002/cam4.3915

**Published:** 2021-05-07

**Authors:** Emmi Kärkkäinen, Sami Heikkinen, Maria Tengström, Veli‐Matti Kosma, Arto Mannermaa, Jaana M. Hartikainen

**Affiliations:** ^1^ School of Medicine Institute of Clinical Medicine, Pathology and Forensic Medicine, and Translational Cancer Research Area University of Eastern Finland Kuopio Finland; ^2^ School of Medicine Institute of Clinical Medicine University of Eastern Finland Kuopio Finland; ^3^ School of Medicine Institute of Biomedicine University of Eastern Finland Kuopio Finland; ^4^ School of Medicine Institute of Clinical Medicine, Oncology, and Cancer Center of Eastern Finland University of Eastern Finland Kuopio Finland; ^5^ Cancer Center Kuopio University Hospital Kuopio Finland; ^6^ Department of Clinical Pathology Kuopio University Hospital Kuopio Finland

**Keywords:** biomarkers, breast cancer, next generation sequencing, non‐coding RNAs, prognostic factor, transcriptomics

## Abstract

Numerous factors influence breast cancer (BC) prognosis, thus complicating the prediction of outcome. By identifying biomarkers that would distinguish the cases with poorer response to therapy already at the time of diagnosis, the rate of survival could be improved. Lately, Piwi‐interacting RNAs (piRNAs) have been introduced as potential cancer biomarkers, however, due to the recently raised challenges in piRNA annotations, further evaluation of piRNAs’ involvement in cancer is required. We performed small RNA sequencing in 227 fresh‐frozen breast tissue samples from the Eastern Finnish Kuopio Breast Cancer Project material to study the presence of piRNAs in BC and their associations with the clinicopathological features and outcome of BC patients. We observed the presence of three small RNAs annotated as piRNA database entries (DQ596932, DQ570994, and DQ571955) in our samples. The actual species of these RNAs however remain uncertain. All three small RNAs were upregulated in grade III tumors and DQ596932 additionally in estrogen receptor negative tumors. Furthermore, patients with estrogen receptor positive BC and higher DQ571955 had shorter relapse‐free survival and poorer BC‐specific survival, thus indicating DQ571955 as a candidate predictive marker for radiotherapy response in estrogen receptor positive BC. DQ596932 showed possible prognostic value in BC, whereas DQ570994 was identified as a candidate predictive marker for tamoxifen and chemotherapy response. These three small RNAs appear as candidate biomarkers for BC, which could after further investigation provide novel approaches for the treatment of therapy resistant BC. Overall, our results indicate that the prevalence of piRNAs in cancer is most likely not as comprehensive as has been previously thought.

## Graphical abstract

We have observed three small RNAs associating with clinical characteristics and patient outcome in breast cancer. The actual species of these RNAs needs further clarification but these results indicate that the dysregulation of piRNAs in cancer is most likely not as general a phenomenon as has been previously thought.

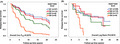

## 1 INTRODUCTION

The identification of molecular factors that associate with breast tumorigenesis and the outcome of breast cancer (BC) patients provides novel potential biomarkers that could enhance diagnostics, disease monitoring and outcome prediction, as well as enable the establishment of new treatment options. Additionally, the identification of dysregulated molecular factors could reveal the mechanisms behind the more aggressive BC sub‐types such as triple negative BC (TNBC), as well as the resistance to therapies. Recently, researchers in the cancer field have expanded their interest into noncoding RNAs (ncRNAs), instead of just focusing on the protein‐coding gene signature.^1^, ^2^ Among the important regulatory small non‐coding RNAs (sncRNAs), the relatively newly discovered Piwi‐interacting RNAs (piRNAs) have been thus far studied mainly in the germline and gonads of model organisms, where their roles in transposon control and genome stability maintenance are well established.^3^, ^4^, ^5^, ^6^ piRNAs are generated from long single‐stranded precursor transcripts that are modified into mature piRNAs by a complex biogenesis machinery.^7^ They function in cooperation with the Piwi‐proteins that bind piRNAs and form complexes that recognize and usually silence their complementary targets either by transcriptionally affecting the epigenetic machinery in the nucleus or by post‐transcriptionally cleaving transcripts in the cytoplasm.^8^, ^9^, ^10^

Even though piRNAs have been considered to be restricted to the germline and gonads, they have been indicated to be present and participate in gene regulation also in the soma, especially in neuronal tissue.^11^, ^12^, ^13^, ^14^ Although the exact mode of function of somatic piRNAs remains unclear, the suggested role of piRNAs and their associated Piwi‐proteins in the soma has raised the question whether they could also affect tumorigenesis. Given their role in the maintenance of genome integrity and the fact that cancer cells and stem cells share characteristics such as indefinite proliferation along with the observed role of Piwi‐proteins and piRNAs in stem cell self‐renewal, they have been considered as intriguing potential players in tumorigenesis.^15^, ^16^, ^17^, ^18^ In fact, due to the observed dysregulation in tumors and associations with growth, invasiveness and outcome of various cancers including BC, the PIWI‐proteins have been suggested as potential biomarkers in cancer diagnostics and prognostics.^19^, ^20^, ^21^, ^22^ Many studies have also suggested piRNAs distinguishing normal from tumorous tissue and their possible prognostic value.^22^, ^23^, ^24^, ^25^, ^26^, ^27^, ^28^, ^29^ However, the functional roles of these reported piRNAs remain widely unknown. Also, a recent study has raised a question whether the observed potential roles of piRNAs in extragonadal somatic tissues, including tumors, are real, as it was shown that databases that are used in annotating the studied piRNAs contain fragments of other ncRNAs leading to the misinterpretation of these fragments as piRNAs.^30^ Thus, it remains elusive, whether piRNAs truly play roles in tumorigenesis.

Here, we have performed a small RNA‐sequencing in the large material of 227 BC and benign breast tissue samples. The vast majority of small RNAs annotated as piRNAs that were observed in our samples were identified as fragments of other ncRNAs, thus questioning the previously reported presence of hundreds of piRNAs in BC by studies that have based their annotations on piRNA databases without precautions. We observed the presence of 19 small RNAs that were annotated in piRNA database, by having at least two counted reads in at least 10 tissue samples. However, only three of these were considered as potential “independent” small RNAs instead of being probable fragments of other RNAs. Nevertheless, all the three small RNAs associated with the clinicopathological features of invasive BC and patient outcome suggesting they could have roles in breast tumorigenesis. Further evaluation of the potential functional roles of these small RNAs would inform on their species and whether they are involved in BC development and progression. Altogether, our results indicate that the abundant piRNA prevalence in breast cancer is most likely not as a general phenomenon as has been previously thought.

## 2 MATERIALS AND METHODS

### 2.1 Sample material

We used the Kuopio Breast Cancer Project (KBCP) material, which has been collected from a genetically homogeneous population in the province of Northern Savo in Eastern Finland (Kuopio University Hospital).^31^, ^32^ The study subjects have given their informed consent for participation in the study. KBCP has been performed in accordance with the Declaration of Helsinki and was approved by the joint ethics committee of the University of Eastern Finland and Kuopio University Hospital (reference numbers 7/89 and 225/2008). It includes fresh‐frozen and archived paraffin‐embedded breast tissue samples, EDTA blood samples and serum, as well as comprehensive background/lifestyle information, full spectrum of updated clinical information from the hospital (KUH) records, including detailed clinical, treatment and follow‐up data extending up to 25 years. For this study, fresh‐frozen tissue material was available from 227 KBCP samples (194 invasive BC tumors, 6 in situ tumors, 22 benign breast tissues and 5 normal breast tissues) (Table S1).

### 2.2 RNA extraction

Fresh‐frozen BC tissue from the primary tumors were obtained during cancer surgery and immediately after resection covered with optimum cutting temperature compound (OTC), cooled in liquid isopentane and liquid nitrogen and stored in −70°C. Total RNA was extracted from the fresh‐frozen BC and benign breast tissue using Ambion mirVana miRNA Isolation Kit (Life Technologies). The concentration of the RNA was assessed with NanoDrop ND‐1000 UV/Vis spectrophotometer (Thermo Scientific), and Qubit 2.0 Fluorometer (Thermo Fisher Scientific) using the Qubit RNA BR (Broad‐Range) Assay Kit (Thermo Fisher Scientific). The quality of total RNA was analyzed using Agilent 2100 Bioanalyzer (Agilent Technologies) with the Agilent RNA 6000 Nano Kit (Agilent Technologies). The average of RIN values of the samples used for library preparation was 8.1 (range 2.2–10.0), 84% of the samples were with RIN ≥7 (92% with RIN ≥5).

### 2.3 Small RNA‐Sequencing

The sequencing libraries for small RNA‐Seq were prepared using the TruSeq Small RNA library prep kit (Illumina, San Diego, CA, USA) according to manufacturer's protocol and sequenced with Illumina MiSeq instrument as 40 nt paired‐end reads, using the first reads per pair for analysis.

### 2.4 Bioinformatic data analysis

Small RNA‐Seq preprocessing consisted of read quality assessment (FastQC, 0.10.1) and adapter trimming (TRIMMOMATIC, v0.39 with essential parameters: ILLUMINACLIP: TruSeqSmallRNA.fa:0:30:10, MINLEN 20, AVGQUAL:30).^33^, ^34^ The human piRNA transcriptome (later: hg38.ncbi.pirna.gtf) was defined by mapping the NCBI piRNA sequences to hg38 using blastn (v2.2.29+; task megablast), discarding all sequences that mapped more than 10 times, and preserving one location for each sequence. The final transcriptome consisted of 25,504 RNAs. Preprocessed reads were aligned to human genome version GRCh38 (primary assembly) using STAR (v2.5.4b), with essential alignment parameters as in Ref. [35] except supplying the above transcriptome “on the fly.” Aligned reads where filtered, using samtools (v1.7), to remove alignments to mitochondrial DNA, Gencode v31 sncRNAs or tRNAs, miRbase v22 primary transcripts for human, and UCSC hg38 RepeatMasker sequences longer than 24 bp, followed by data conversions (e.g., for visualization (samtools, IGVtools)). Gene‐wise counts of primary alignments that overlapped a transcriptome member by at least 80% of the read length (but at least 15nt) were collected using the R function Rsubread::featureCounts (v1.30.9) with essential parameters: minOverlap=15, fracOverlap=0.80, largestOverlap=TRUE, countMultiMappingReads=TRUE, minMQS=1, primaryOnly=TRUE. For uses other than differential gene expression (DEG) analysis, read counts were normalized using R function DESeq2::varianceStabilizingTransformation in “blind” mode (v1.22.1).^36^ To identify technical bias, quality control and exploration were performed (multidimensional scaling, principal component analysis, and unsupervised hierarchical clustering) in R/Bioconductor ^37^ ; no bias assignable to, for example, library preparation batch or sequencing run was found.

### 2.5 Statistical analyses

Statistically differentially expressed (DE) RNAs in hg38.ncbi.pirna.gtf were identified using R package DESeq2 (v1.22.1), using Wald as test type, FDR for *P*‐value adjustment and the R function DESeq2::lfcShrink for shrinking fold changes of low expressed RNAs. In the DEG analyses the following BC sub‐types and clinical variables were tested: invasive BC versus benign breast tissue, estrogen receptor (ER) negative versus ER positive BC, progesterone receptor (PR) negative versus PR positive BC, HER2 negative versus HER2 positive BC, node positive versus node negative, TNBC versus luminal BC, HER2‐type versus TNBC, HER2‐type versus non‐HER2‐type, TNBC versus luminal A, TNBC versus luminal B, TNBC versus non‐TNBC, luminal B versus A, non‐luminal versus luminal, tumor grade versus benign, BC sub‐types versus benign, tumor histology versus benign, malign versus benign, tumor stage versus benign and tumor size versus benign. Tumor grades, sizes, histology types and stages were compared pairwise within each variable.

Only cases with invasive, local disease (i.e., excluding in situ cases and those with metastases at diagnosis) were included in the survival analyses for relapse‐free survival (RFS), BC‐specific survival (BCSS) and overall survival (OS). RFS was defined as the time between the date of BC diagnosis and the date of recurrence. BCSS was defined as the time between the date of BC diagnosis and the date of death due to BC, and OS as the time between the date of BC diagnosis and the date of death due to any cause. For the analysis of each small RNA with enough aligned reads to be considered as being present, cases were divided into quartiles by the normalized read count, Q1 denoting the quartile with the lowest read count. All survival analyses were performed, for a given RNA, by comparing each other quartile against Q1. Univariate survival analyses were performed using the R function survival::coxph that implements the Cox's proportional hazards model. Multivariate survival analyses providing the hazard ratios (HR) and confidence intervals (CI) for death (for BCSS and OS) or recurrence (for RFS) were performed using the Cox's proportional hazards model in a forward stepwise manner implemented in R function MASS::stepAIC v7.3–51.1. The additional data included in the multivariate survival analyses as covariates were, for clinical parameters, tumor grade, tumor histology, tumor size, ER status, PR status, HER2 status and age at diagnosis, and for treatment parameters, radiotherapy (RT) (yes/no), adjuvant chemotherapy (CT) (yes/no) and adjuvant endocrine therapy (ET) (yes/no). In addition to all cases with invasive local disease, the multivariate survival analyses were also performed separately for specific patient groups, defined by the received treatment as follows:

1. “RT‐treated cases” (cases with invasive local BC, has received RT): Adjuvant CT (y/n), adjuvant ET (y/n) and clinical data included in the analysis as covariates.
2. “Tamoxifen‐treated cases” (cases with invasive local, ER positive BC, has received adjuvant tamoxifen, but no adjuvant CT): RT (y/n) and clinical data included in the analysis as covariates.
3. “RT only cases” (cases with invasive local BC, has received RT, but no adjuvant CT or ET): Clinical data included in the analysis as covariates.
4. “Adjuvant CT‐treated cases” (cases with invasive local BC, has received adjuvant CT, but no adjuvant ET): RT (y/n) and clinical data included in the analysis as covariates.
5. “Surgery‐only cases” (cases with invasive local BC, has not received adjuvant ET, CT or RT): Clinical data included in the analysis as covariates.

Kaplan–Meier survival plots for both univariate and multivariate analyses were generated using SPSS (v25).

### 2.6 qRT‐PCR

qRT‐PCR was performed to confirm the presence of DQ570994, DQ596932, DQ571955 in a subset of the breast tissue samples (n = 44). The reverse transcription (RT) was performed according to the instructions of the manufacturer using 10 ng of total RNA, the TaqMan MicroRNA Reverse Transcription Kit (Thermo Fisher Scientific, Waltham, MA, USA) and specific RT primers obtained from the Custom TaqMan Small RNA Assays (Thermo Fisher Scientific) for DQ570994, DQ596932, DQ571955, and the endogenous control U6. qPCR was performed in triplicate reactions using the Custom TaqMan Small RNA Assays for DQ570994, DQ596932, DQ571955, and U6 (Thermo Fisher Scientific), TaqMan Universal PCR Master Mix II no UNG (Thermo Fisher Scientific), and the LightCycler 96 Instrument (Roche) according to manufacturers’ instructions.

The relative expression for each small RNA was calculated from the Cq values using the ΔΔCq method. Pearson correlation was used for calculating the correlation of the relative expression in log2 scale (qRT‐PCR) with normalized read count in log2 scale (small RNA‐seq).

## 3 RESULTS

### 3.1 The presence of three small RNAs aligning to piRNA locations were observed in breast tissue

The short RNA‐seq generated 548.7 million raw reads for the 227 sequenced samples, ranging from 1.12 to 6.62 million per sample. Preprocessing decreased the total to 479.4 million and the per‐sample range to 0.95 to 4.28 million. Since the short RNA‐seq data inherently include reads for several short RNA classes, it is not surprising that even without further filtering the total read set that counted to piRNA database entries became only 18.7 million, ranging from 31,765 to 234,850 reads per sample. However, due to the recently identified biases in current piRNA databases, we excluded from further analysis all alignments to chrM, other known sncRNAs and DNA repeats longer than 24nt.^30^ This reduced the total read count over all samples to only 158,310 reads, ranging from 144 to 4498 reads per sample. It should be noted that we only counted primary alignments in which the read overlapped the genomic location of the piRNA database entry by at least 80%. After the thorough filtering, there remained 1371 piRNA database entries with at least one counted read in any of the 227 samples. However, only 19 of these were considered to be present at a sufficient level, by having at least two counted reads in at least 10 samples. The aligned read stacks at the loci of these 19 small RNAs were further inspected in a genome browser for well‐defined uniformity and direct overlap with the piRNA sequence location. This suggested that only three of these small RNAs have the potential to be piRNAs or other type of small RNAs; DQ570994 (piR‐31106), DQ571955 (piR‐40067), and DQ596932 (piR‐34998) (Figures S1‐S3). In addition, for two RNAs [DQ593736 (piR‐33848) and DQ595292 (piR‐61404)] among those that were considered “not present at a sufficient level,” but that still had at least one read in at least 25 samples, we observed a uniform read stack directly on the piRNA location (Figure S4). The genomic locations of these five small RNAs and the overlapping genes are listed in Table S2, and their normalized read counts for all 227 sequenced KBCP samples are given in Table S3, together with selected clinical characteristics. Notably, DQ571955 and DQ595292 harbor the 5’ uridine (U) that is preferred by PIWI‐proteins upon binding. The presence of DQ570994, DQ596932, and DQ571955 was confirmed in qRT‐PCR analysis, although for DQ571955 the levels were close to the detection limit of qRT‐PCR (Cq 31–35). Moreover, a statistically significant correlation was observed for DQ570994 and DQ596932 between qRT‐PCR and small RNA‐seq measurements (Figure S5).

### 3.2 The three small RNAs DQ571955, DQ596932, and DQ570994 associate with the clinicopathological features of BC

In the DEG analyses including all invasive BC cases, the three observed small RNAs (DQ570994, DQ596932, and DQ571955) associated with the clinicopathological features of BC (Table 1). They were upregulated in grade III tumors (n=69) when compared to grade I tumors (n = 32) (Table 1, Figures S6A–C), and DQ596932 also when compared to grade II tumors (n = 93) (Table 1, Figure S6B). Additionally, DQ596932 was upregulated in ER negative tumors (n = 56) compared to ER positive tumors (n = 137), and accordingly in non‐luminal BC compared to luminal BC (Table 1, Figures S6D,E). DQ596932 also showed a trend toward its upregulation in TNBC (n = 33), when compared to luminal BC (n = 137), although the adjusted *P*‐value was not statistically significant (Figure S7A). When only the cases with luminal A tumors were included in the analysis, the differential expression in comparison to TNBC was more pronounced (Figure S7B). DQ571955 in turn seemed to be present exclusively in invasive BC, when compared to benign breast tissue, even though the association again was not statistically significant (likely due to a couple of outliers in the benign group) (Figure S8A). The difference in the presence of DQ570994 and DQ596932 between invasive breast cancer and benign breast tissue was not as pronounced as for DQ571955 (Figure S8B, C). Furthermore, DQ570994 was upregulated in grade III tumors compared to grade I and II tumors also when the analyses were adjusted for ER status, which indicates that the association is independent of the ER status (Table 1, Figure S9A,B). When the DEG analyses were restricted to cases with invasive local BC, similar results were otherwise obtained for all three small RNAs except for DQ571955, which was no longer significantly DE in the comparison of grade III vs. grade I tumors (Table 1).

**TABLE 1:** The small RNAs annotated as piRNA database entries significantly associated (*p*_adj_ < 0.05) with tumor characteristics

		Grade III vs. I (ref)		Grade III vs. II (ref)		ER‐ vs. ER+ (ref)		Non‐ luminal vs. luminal (ref)	
Sample set	Accession^a^	Log_2_(FC)^b^	*p*_adj_ ^c^	Log_2_(FC)^b^	*p*_adj_ ^c^	Log_2_(FC)^b^	*p*_adj_ ^c^	Log_2_(FC)^b^	*p*_adj_ ^c^
Invasive BC									
	DQ570994	1.328	0.0001	0.684	ns	0.061	ns	0.061	ns
	DQ596932	1.368	0.0018	0.997	0.0034	0.941	0.0361	0.941	0.0361
	DQ571955	1.253	0.0234	0.438	ns	0.605	ns	0.605	ns
Adjusted by ER status									
	DQ570994	1.673	8.68e^−06^	0.958	0.0121	NA	NA	NA	NA
Invasive local BC									
	DQ570994	1.325	0.0002	0.708	ns	0.103	ns	0.103	ns
	DQ596932	1.356	0.0025	1.011	0.0033	0.997	0.0135	0.997	0.0135
Adjusted by ER status									
	DQ570994	1.603	3.88e^−05^	0.932	0.0264	NA	NA	NA	NA

Analyses were done in all invasive BC cases and in cases with invasive local BC for the comparisons of grade III tumors vs. grade I tumors and grade II tumors, ER negative tumors vs. ER positive tumors and non‐luminal BC vs. luminal BC.

Abbreviations: BC, breast cancer; ER, estrogen receptor; FC, Fold change; NA, Not applicable; ns, statistically nonsignificant (*p*_adj_ > 0.05); *p*_adj_, adjusted *p*‐value; Ref, Reference category.

^a^ NCBI GenBank accession number.

^b^ The Log_2_‐transformed fold change for the differential expression.

^c^ The FDR‐adjusted *p*‐value for the fold change.

### 3.3 DQ596932 associates with BC prognosis, whereas DQ571955 and DQ570994 predict response to therapy

**FIGURE 1:**
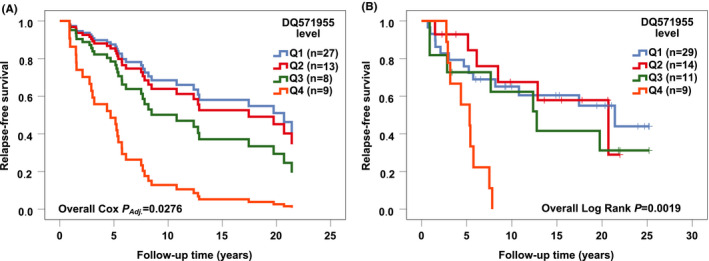
The association of DQ571955 with relapse‐free survival (RFS) in the estrogen receptor (ER) positive radiotherapy‐treated cases. (A) The highest quartile (Q4) of DQ571955 significantly associated with poorer RFS [*p *= 0.0012, HR (CI 95%) = 5.21 (1.92–14.16)], when compared to the lowest quartile (Q1) in the ER positive radiotherapy‐treated cases (n = 57) in the Cox multivariate analysis. (B) Kaplan‐Meier plot showing the highest quartile (Q4) of DQ571955 significantly associated with poorer RFS [Overall Log Rank *p *= 0.0019, for Q4 *p *= 0.0009, HR (CI 95%) = 4.76 (1.90–11.94)] in the ER positive radiotherapy‐treated cases (n = 63) in the univariate survival analysis

**FIGURE 2:**
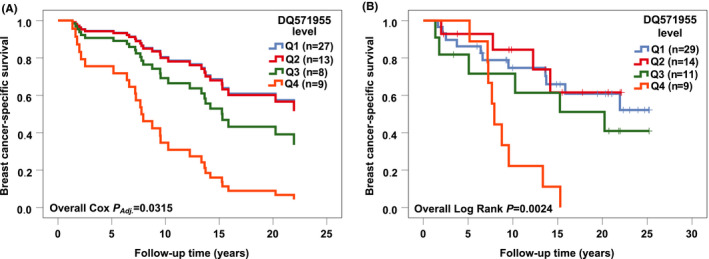
The association of DQ571955 with breast cancer‐specific survival (BCSS) in the estrogen receptor (ER) positive radiotherapy‐treated cases. (A) The highest quartile (Q4) of DQ571955 significantly associated with poorer BCSS [*p *= 0.0003, HR (CI 95%) = 6.84 (2.44–19.19)], when compared to the lowest quartile (Q1) in the ER positive radiotherapy‐treated cases (n = 57) in the Cox multivariate analysis. (B) Kaplan‐Meier plot showing the highest quartile of DQ571955 significantly associated with poorer BCSS [Overall Log Rank *p *= 0.0024, for Q4 *p *= 0.0015, HR (CI 95%) = 4.47 (1.77–11.28)] in the ER positive radiotherapy‐treated cases (n = 63) in the univariate survival analysis

In the Cox multivariate survival analyses, DQ571955 was identified as a potential predictive marker for RT response in ER positive BC as it associated with patient outcome only in the ER positive RT‐treated cases (n = 57); the highest quartile of DQ571955 associated with poorer RFS and BCSS, when compared to the lowest quartile (Table 2, Figures 1A and 2A). The observed associations were statistically significant also in the univariate survival analyses (Figures 1B and 2B).

**TABLE 2:** The small RNAs annotated as piRNA database entries significantly associated with patient outcome in the Cox multivariate survival analyses in cases with invasive local BC

Accession^a^	Survival type	*p*_Overall_ ^b^	*p*_adj_ ^c^	*p* ^d^			HR (CI 95%)^e^		
				Q2	Q3	Q4	Q2	Q3	Q4
RT‐treated ER positive cases (n = 57)									
DQ571955	RFS	**0.0055**	**0.0276**	0.5310	0.7193	**0.0012**	1.38 (0.50–3.77)	1.22 (0.41–3.66)	**5.21 (1.92–14.16)**
	BCSS	**0.0095**	**0.0315**	0.7298	0.5551	**0.0003**	1.23 (0.38–4.04)	1.42 (0.44–4.57)	**6.84 (2.44–19.19)**
DQ596932	OS	**0.0286**	**0.0375**	0.1113	**0.0067**	0.0540	0.46 (0.17–1.20)	**0.20 (0.06–0.64)**	0.43 (0.18–1.01)
Tamoxifen‐treated ER positive cases (n = 31)									
DQ570994	OS	**0.0498**	0.0692	**0.0131**	**0.0124**	0.1300	**7.61 (1.53–37.85)**	**8.16 (1.57–42.31)**	3.20 (0.71–14.44)
DQ596932	OS	0.0661	0.0703	0.5800	**0.0422**	**0.0063**	0.64 (0.13–3.10)	**0.11 (0.01–0.93)**	**0.16 (0.04–0.60)**
Surgery only, ER positive cases (n = 53)									
DQ596932	BCSS	0.0527	0.0843	0.2315	0.2042	**0.0171**	4.41 (0.39–50.21)	4.16 (0.46–37.48)	**15.65 (1.63–150.06)**
	OS	0.0861	0.0967	0.6899	0.1006	**0.0343**	0.82 (0.30–2.21)	0.47 (0.19–1.16)	**3.00 (1.08–8.28)**
Adjuvant CT‐treated cases (n = 27)									
DQ570994	RFS	0.0635	0.0877	**0.0281**	0.8435	0.1064	**5.36 (1.20–24.03)**	0.80 (0.08–7.68)	2.97 (0.79–11.15)

Clinical data (histology, tumor size, grade, age at diagnosis, nodal‐, ER^‡‡^, PR and Her2 status) and treatment data (RT^§§^ or CT^¶¶^ and ET yes/no) were included as covariates in the analyses in addition to the RNA quartiles. Statistically significant associations (*p*_Overall_ < 0.05 and/or Q vs Q1 *p *< 0.05) are shown in bold.

Abbreviations: BC, breast cancer; BCSS, breast cancer‐specific survival; CI, confidence interval; CT, chemotherapy; ER, estrogen receptor; HR, hazard ratio; OS, overall survival; *p*_adj_, adjusted *p*‐value; RFS, relapse‐free survival; RT, radiotherapy.

^a^ NCBI GenBank accession number.

^b^ The overall *p*‐value for the significance of difference in patient outcome between RNA quartiles calculated using the Cox proportional hazards model.

^c^ The FDR‐adjusted overall *p*‐value (*p*_Overall_).

^d^ The *p*‐value for the significance of difference in patient outcome between RNA quartile compared to Q1 calculated using the Cox proportional hazards model.

^e^ For a given survival type, the HR and 95% CI of BC recurrence (RFS), death due to breast cancer (BCSS), and death due to any cause (OS) calculated using the Cox proportional hazards model.

‡‡ ER status was not included as a covariate in the analyses restricted to ER positive cases.

§§ RT (yes/no) was included as a covariate in the analyses restricted to the ER positive cases who had received tamoxifen therapy and in the analyses restricted to the cases who had received CT.

¶¶ CT and ET (yes/no) were included as covariates only in the analyses restricted to the ER positive cases who had received RT.

In the ER positive RT‐treated cases, the quartile Q3 of DQ596932 associated with better OS compared to Q1 (Table 2, Figure S10A). Overall, even when the association with better OS with Q2 and Q4 did not reach statistical significance, the lowest quartile clearly associated with poorer OS compared to higher quartiles (Table 2, Figure S10A). In the univariate survival analysis, the association between DQ596932 level and OS was not statistically significant (Figure S10B).

DQ570994 was identified as a possible predictive marker for tamoxifen response as multivariate analyses associated it with patient outcome in the tamoxifen‐treated cases (Table 2), but not in the evaluation of all invasive cases with local disease or in the cases treated with surgery only (data not shown). The quartiles Q2 and Q3 of DQ570994 associated with poorer OS in the tamoxifen‐treated cases (n=31) (Table 2, Figure S11A). The highest quartile seemed to associate with better OS than Q2 and Q3, but this might be due to the low number of cases in the analysis (Figure S11A). In the univariate survival analysis, the association of DQ570994 quartile Q2 with OS was statistically significant in the tamoxifen‐treated cases (n = 35). However, while the overall association was not significant, the outcome tends to be better for the cases in the lowest quartile group (Figure S11B).

Also, the higher quartiles of DQ596932 associated with better OS in the tamoxifen‐treated cases (n = 31), even though the overall *P*‐value in these analyses did not reach statistical significance (Table 2, Figure S12A). Although the number of cases involved here was low, the difference in the probability of death (due to any cause) is obvious between the higher and the lower quartiles. The higher DQ596932 quartiles tended to associate with better OS also in the univariate survival analysis (n = 35) (Figure S12B).

Additionally, in the ER positive, surgery‐only cases, the highest DQ596932 quartile associated with poorer BCSS and OS (Table 2, Figures S13A and S14A) in the multivariate analysis (n = 53); however, the overall *P*‐values were not statistically significant (Table 2). The statistically significant association of the highest DQ596932 quartile with BCSS was also seen in the univariate survival analysis (Figure S13B) but not in the analysis for OS (Figure S14B). Taken together, DQ596932 associated with patient outcome in the ER positive cases who had received RT, tamoxifen or only surgery suggesting that it might have potential as a prognostic marker for BC in general, and not in a specific treatment group only.

DQ570994 may also have potential as a predictive marker for CT response as it associated with patient outcome in the adjuvant CT‐treated cases (Table 2) but not in all invasive cases with local disease or in the surgery‐only cases (data not shown). The overall *P*‐values in the multivariate survival analysis did not reach statistical significance, but the quartile Q2 of DQ570994 associated with poorer RFS in the adjuvant CT‐treated cases (n = 27), and the lowest quartile clearly associated with longer RFS, suggesting that it may have an effect on the response to CT (Table 2, Figure S15A). The number of cases available for this multivariate analysis was low (n = 27), but the difference in the occurrence of a relapse was obvious between the quartiles. In the univariate survival analysis, the association of DQ570994 with RFS was not statistically significant (Figure S15B).

In the RT‐only cases (n = 24), the higher quartiles of DQ596932 associated with better OS [*p* = 0.0059, HR (CI 95%) = 0.01 (3.6e^−04^–0.26) for Q3, and *p *= 0.0095, HR (CI 95%) = 3.6e^−04^ (8.8e^−7^–0.14) for Q4]. However, the overall adjusted *p*‐value was not statistically significant (0.1002) and in this multivariate analysis the number of samples was very low, which also contributed to the wide CI, so this result should be interpreted with caution (further data not shown).

## 4 DISCUSSION

In addition to the widely studied sncRNAs such as miRNAs, the fairly recently discovered piRNAs have been thought to provide a novel viewpoint for investigating the processes involved in cancer development and progression. Even though the levels of piRNAs in the germline and gonads are known to exceed those described in other somatic tissues, piRNAs have been suggested to participate in tumorigenesis as potential cancer biomarkers due to their reported dysregulation in cancer and association with the outcome of various cancer types.^23^, ^24^

Interestingly, recently it was pointed out that most of the present small RNAs in extragonadal somatic tissues that are annotated as piRNAs and that map to the same genomic location as other ncRNAs are not functional piRNAs but fragments of these other ncRNAs.^30^ Supporting this observation, the vast majority of the small RNAs observed in our samples that are annotated as piRNA database entries were in fact identified as fragments of other ncRNAs. After careful evaluation, we observed the presence of only three small RNAs in our samples (DQ570994, DQ571955, and DQ596932), that were not obviously derived from other (small) RNA species, one of which (DQ571955) harbors the characteristic Uridine at its 5’ end.^38^ Our finding supports another recent study, where Genzor and co‐workers investigated in 2019, whether PIWI‐proteins and piRNAs are involved in tumorigenesis in colon cancer cells.^39^ The authors observed piRNAs to be absent after the removal of reads that align to other ncRNAs.^39^ These observations imply that the majority of the previously observed aberrant piRNA levels in cancer could in fact arise from the misinterpretation of the fragments of other ncRNAs as piRNAs, and thus question their prominent involvement in tumorigenesis. Apart from these three small RNAs, we did not detect any of the previously described piRNAs in our dataset.^24^

The small RNAs present in our dataset (DQ570994, DQ571955, and DQ596932) associated with tumors of higher grade, a strong prognostic determinant, and hence offer candidates for prognostic and therapeutic markers for BC. The observed association with DQ596932 upregulation with negative ER status might be an indication of the TNBC sub‐type. However, since there are multiple factors contributing to the BC sub‐types, the potential TNBC association requires further confirmation. All the three small RNAs associated also with patient outcome. The biomarker potential of DQ571955 and DQ596932 seem to relate to the ER status as they associated with patient outcome only in the ER positive patient groups, and with the tumor grade only in the analyses not adjusted for ER status. The low number of cases available for the analyses restricted to certain treatment groups and the lack of previous reports concerning the possible predictive role of the small RNAs identified here, point toward the need of further research to confirm their prognostic potential. Supporting our results regarding the observed associations of the three small RNAs with the grade III tumors, the upregulation of DQ571955 and DQ570994 in gastric adenocarcinoma and the exclusive presence of DQ596932 in adenocarcinoma compared to normal gastric tissue have been previously observed.^26^ Additionally, DQ570994 has been shown to be upregulated in both BC cells and tumors, but curiously, DQ571955 has been shown to be downregulated in BC cells.^40^ In another study by Martinez et al., the association between DQ571955 and survival in BC was reported; however, the nature of the association was not specified in their report.^25^ Furthermore, consistent with our findings, another study has described the upregulation of DQ570994 and its association with poorer OS and functional oncogenic roles in colorectal cancer.^41^ This small RNA was also observed to be upregulated in several cancers in the TCGA datasets, implicating that DQ570994 may have a role in tumorigenesis in general.^41^

Altogether, DQ570994, DQ571955, and DQ596932 were statistically significantly associated with tumor characteristics and patient outcome in our study supporting their possible involvement in BC. They each map only to a single genomic location that is also devoid of other ncRNAs. Furthermore, the reads formed a uniform stack directly on the mapped locations, which stand out from the background, suggesting that these three RNAs are not just fragments of other RNA species. However, in the light of the recent observations concerning the reliability of piRNA annotations in the context of non‐gonadal tissue, we acknowledge that caution must be taken when evaluating whether these piRNA database entries are actually piRNAs. Notably, DQ596932 and DQ570994 do not harbor the 5’ U that is a characteristic of primary piRNAs.^38^ Thus, further investigation of these potential small RNAs is needed to confirm their actual species. Using available online tools (gene expression microarray data in Kaplan‐Meier plotter and ROC plotter) we also looked for evidence for the association of the expression of the small RNAs’ host genes with patient outcome in BC.^42^, ^43^, ^44^, ^45^ Survival data (RFS and OS) was available for tamoxifen‐treated (Kaplan‐Meier plotter), chemotherapy‐treated (ROC plotter), and for surgery‐only (Kaplan Meier plotter) cases but not for the cases who had received radiotherapy. Only two statistically significant results from the online data paralleled our results. First the higher expression of the DQ596932 host gene *FLII* associated with better OS in the ER positive tamoxifen‐treated cases (*p *= 0.0015, FDR 20%), as did DQ596932 in our data. Second, the higher levels of DQ570994 host gene *ABCA2* associated with poorer RFS in the ER positive cases who had received chemotherapy (*p *= 0.013, FDR<5%), as did DQ570994 in our data. However, for example, *ABCA2* expression did not associate with OS in the tamoxifen treated ER positive cases while DQ570994 did. Thus, clear evidence is lacking that the survival associations we observed for the three small RNAs would only be a direct consequence of the altered expression of their host genes.

To our knowledge, only a few studies have been able to demonstrate the potential functional role of a piRNA in tumorigenesis. Just recently, a pseudogene‐derived somatic piR‐FTH1 was demonstrated to repress the corresponding endogenous *ferritin heavy chain 1* (*FTH1*) gene through the interaction with HIWI2 and HILI proteins in TNBC cells.^46^ Additionally, piR‐932 has been indicated to interact with HILI in BC stem cells in which they both were upregulated and HILI expression was linked to increased Latexin methylation.^21^ Neither piR‐FTH1 nor piR‐932 was present in our samples. A few other studies have also reported functional mechanisms of piRNAs in cancer but they lack validation of the interaction with PIWI‐proteins and two of the reported piRNAs align with snoRNAs.^47^, ^48^, ^49^, ^50^, ^51^ Although these findings shed light on the possible functional roles of individual piRNAs in cancer, whether and how the majority of the observed dysregulated piRNAs in cancer affect tumorigenesis remain unknown. Since sncRNAs have been shown to target their host genes, we investigated whether the host genes of the candidate biomarker small RNAs identified here could serve as target genes based on full sequence complementarity. As these small RNAs do not have a reverse fully complementary sequence in the coding region of their host genes or the corresponding endogenous gene of host pseudogenes, or anywhere else in the transcriptome or genome except for the expected single, full length, 100% reverse matches to chromosomal sequences, their possible target genes may be targeted through partial complementary and could not be pinpointed here.

In conclusion, to date, the functional role of piRNAs in cancer has been suggested for a few potential piRNAs indicating that individual piRNAs could indeed have roles in tumorigenesis, although many studies which have implicated the existence of piRNAs in cancer and their potential diagnostic and prognostic value, have relied on piRNA databases that seem the be contaminated with fragments of other (s)ncRNAs and thus distorting the evidence of the presence of piRNAs in cancer tissues. We observed the presence of three small RNAs in BC and their association with the clinicopathological features and patient outcome of BC in the large material of 227 tissue samples. Although we could not adequately confirm the species of these small RNAs, they appear as candidate prognostic or predictive markers for BC. This demonstrates that further research is needed with additional, even larger sample cohorts including broad clinical data and thorough filtering to evaluate the prognostic and therapeutic value of piRNAs. Furthermore, the functional evaluation of piRNAs is crucial for gaining evidence of their assumed involvement in cancer and of the mechanisms through which they may take part in tumorigenesis.

## CONFLICT OF INTEREST

None declared.

## AUTHOR CONTRIBUTIONS

JMH and AM conceived and designed the study. JMH supervised and participated in the implementation of the experiments. JMH, AM, VMK, and MT acquired data. SH carried out the bioinformatic and the statistical analyses. EK collected the results and drafted the manuscript. EK, JH, SH and MT interpreted the results and reviewed and revised the manuscript. All authors approved the final manuscript.

## ETHICAL APPROVAL STATEMENT

KBCP and this study has been performed in accordance with the Declaration of Helsinki and was approved by the joint ethics committee of the University of Eastern Finland and Kuopio University Hospital (reference numbers 7/89 and 225/2008).

## Supporting information

Fig S1‐S4

Fig S5

Fig S6‐S9

Fig S10‐15

Table S1

Table S2

Table S3

## Data Availability

The sequencing data generated and analyzed during the current study are not publicly available, because they contain information that could compromise research participant privacy and consent but are available from the corresponding author [JMH] on reasonable request.
