# Supplementary material for: The debatable presence of PIWI‐interacting RNAs in invasive breast cancer
Source: Cancer Med. 2021 May 7;10(11):3593–603. doi: 10.1002/cam4.3915 (PMC8178507; doi:10.1002/cam4.3915)
Supplement: Supplementary file 1 — Fig S1‐S4 [file CAM4-10-3593-s001.pdf]

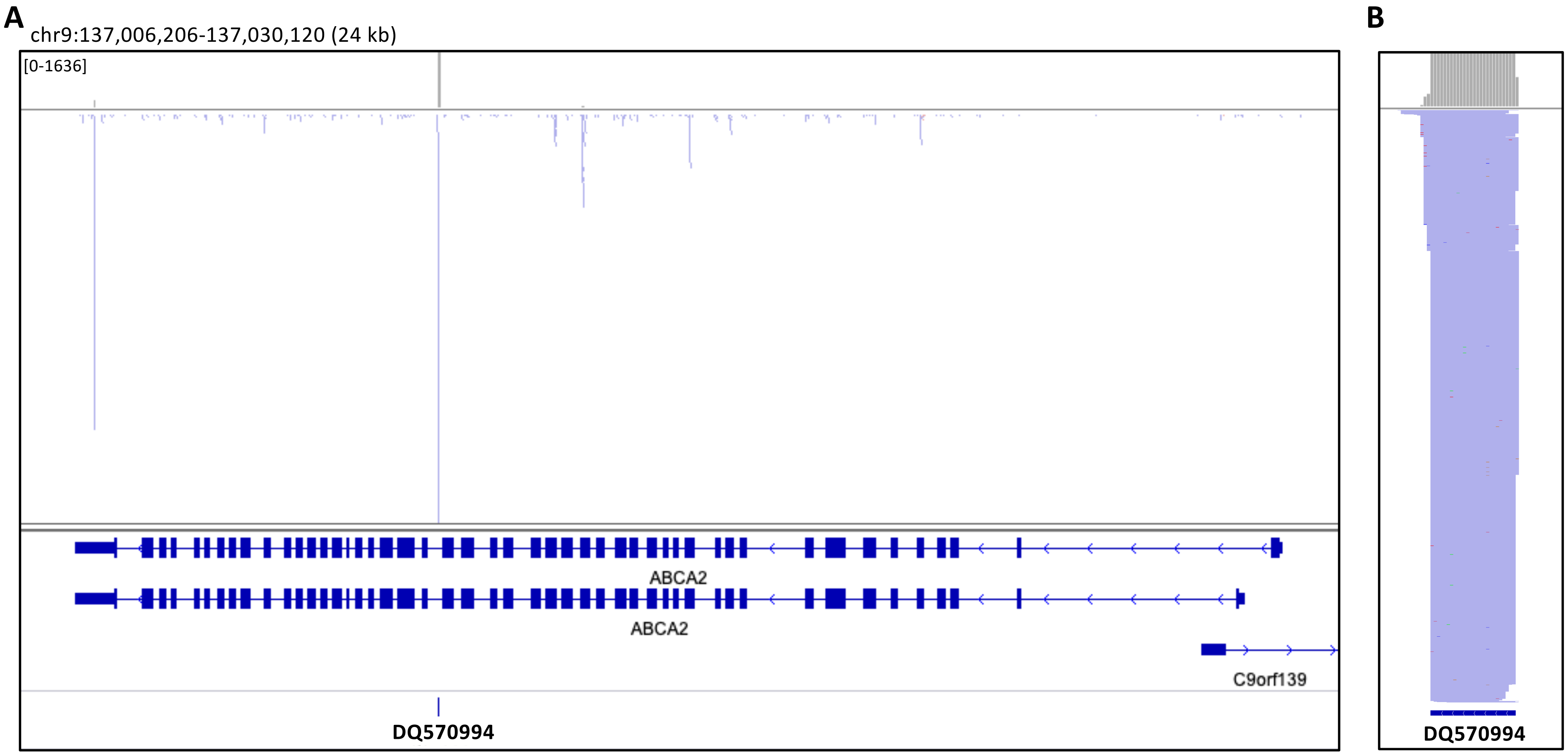


Supplementary Figure S1: The genomic context (**A**) and the uniform stack (**B**) of aligned and filtered short RNA-seq reads combined from all 227 BC samples at the location of DQ570994. In both **A** and **B**, the top two panels display the read coverage in gray [with the range of the per-basepair read count], and the accumulation of reads aligning to the reverse (in blue) or the forward (in red) strand. The location of DQ570994 is indicated at the bottom. In **A**, the genomic coordinates of the displayed region (and size) are given above the graph. For clarity, the high read stack for DQ570994 is shown only partly. In **B**, colored dots within the reads mark mis-matches between the individual reads and the human reference genome.

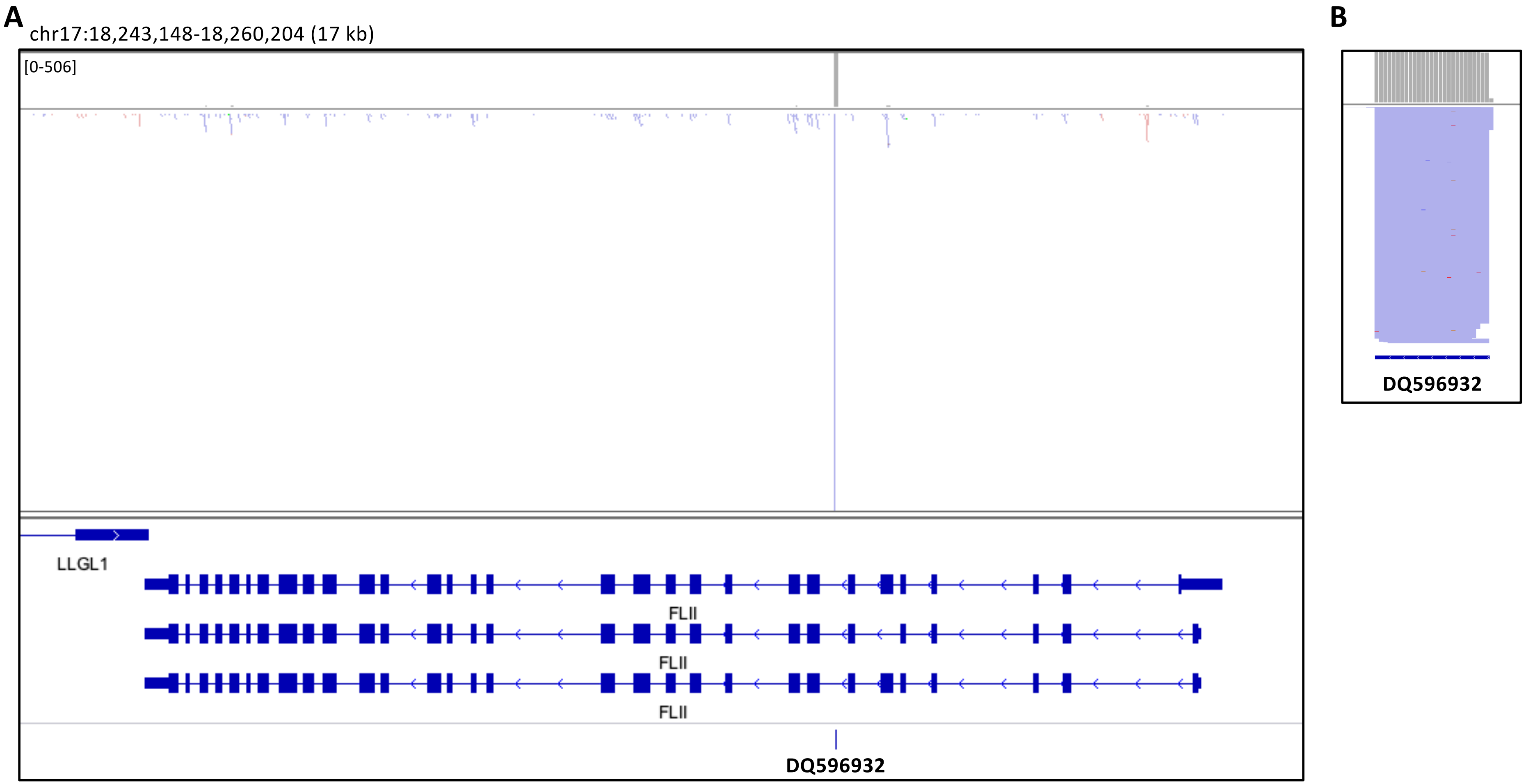


Supplementary Figure S2: The genomic context (**A**) and the uniform stack (**B**) of aligned and filtered short RNA-seq reads combined from all 227 BC samples at the location of DQ596932. In both **A** and **B**, the top two panels display the read coverage in gray [with the range of the per-basepair read count], and the accumulation of reads aligning to the reverse (in blue) or the forward (in red) strand. The location of DQ596932 is indicated at the bottom. In **A**, the genomic coordinates of the displayed region (and size) are given above the graph. For clarity, the high read stack for DQ596932 is shown only partly. In **B**, colored dots within the reads mark mis-matches between the individual reads and the human reference genome.

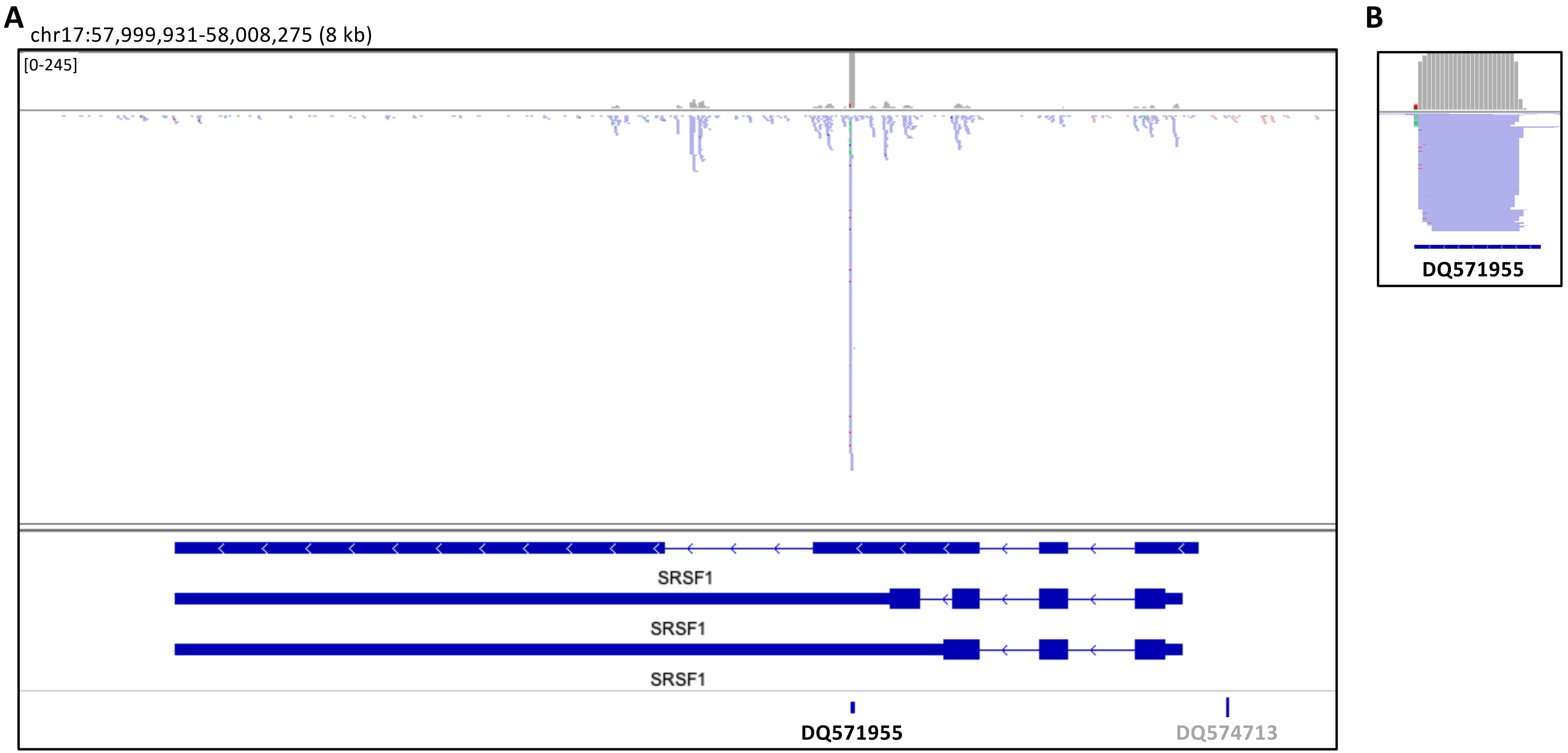


Supplementary Figure S3: The genomic context (**A**) and the uniform stack (**B**) of aligned and filtered short RNA-seq reads combined from all 227 BC samples at the location of DQ571955. In both **A** and **B**, the top two panels display the read coverage in gray [with the range of the per-basepair read count], and the accumulation of reads aligning to the reverse (in blue) or the forward (in red) strand. The location of DQ571955 is indicated at the bottom. In **A**, the genomic coordinates of the displayed region (and size) are given above the graph. The locus also hosts another, non-expressed piRNA database entry (DQ574713, in gray). In **B**, colored dots within the reads mark mismatches between the individual reads and the human reference genome.

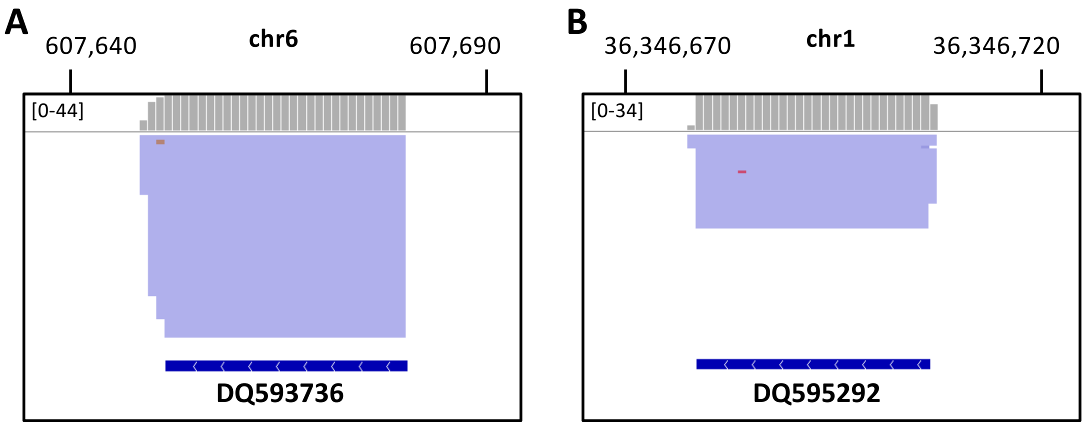


Supplementary Figure S4: The uniform stacks of aligned and filtered short RNA-seq reads combined from all 227 BC samples at the locations of **A**, DQ593736 and **B**, DQ595292. The top panel displays the read coverage in gray [with the range of the per-basepair read count]. The aligned reads are shown in blue for the reverse strand and in red for the forward strand. The locations and accession numbers of the piRNA database entries are indicated at the bottom. Colored dots within the reads mark mismatches between the individual reads and the human reference genome.
