# Supplementary material for: The debatable presence of PIWI‐interacting RNAs in invasive breast cancer
Source: Cancer Med. 2021 May 7;10(11):3593–603. doi: 10.1002/cam4.3915 (PMC8178507; doi:10.1002/cam4.3915)
Supplement: Supplementary file 2 — Fig S5 [file CAM4-10-3593-s002.pdf]

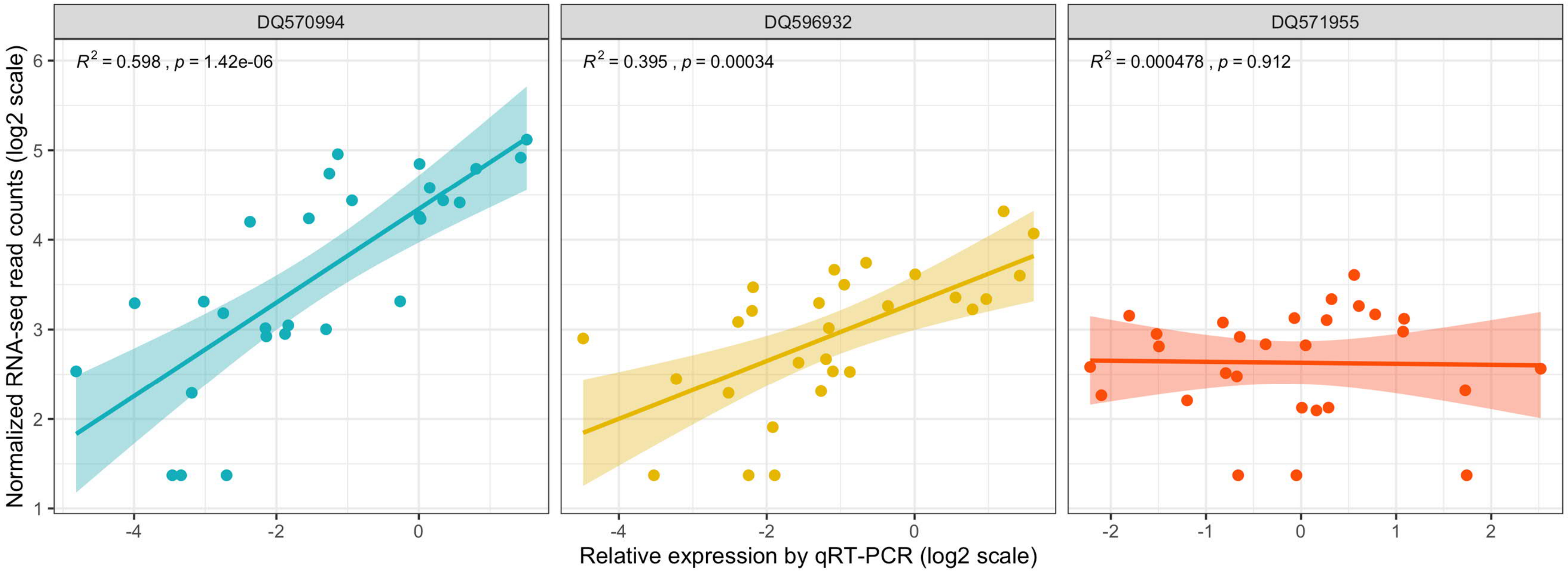

Supplementary Figure S5. Validation of RNA-seq results using qRT-PCR. Relative expression measured using qRT-PCR on x-axis is plotted against vst-normalized RNA-seq read counts on y-axis, both in log2 scale, for DQ570994 (left), DQ596932 (middle) and DQ571955 (right). The plots include a linear regression line with the surrounding colored area representing 95% confidence interval. The Pearson correlation R2 and two-sided *P*-values are given at the top left corners of each plot.
