# Supplementary material for: The debatable presence of PIWI‐interacting RNAs in invasive breast cancer
Source: Cancer Med. 2021 May 7;10(11):3593–603. doi: 10.1002/cam4.3915 (PMC8178507; doi:10.1002/cam4.3915)
Supplement: Supplementary file 3 — Fig S6‐S9 [file CAM4-10-3593-s004.pdf]

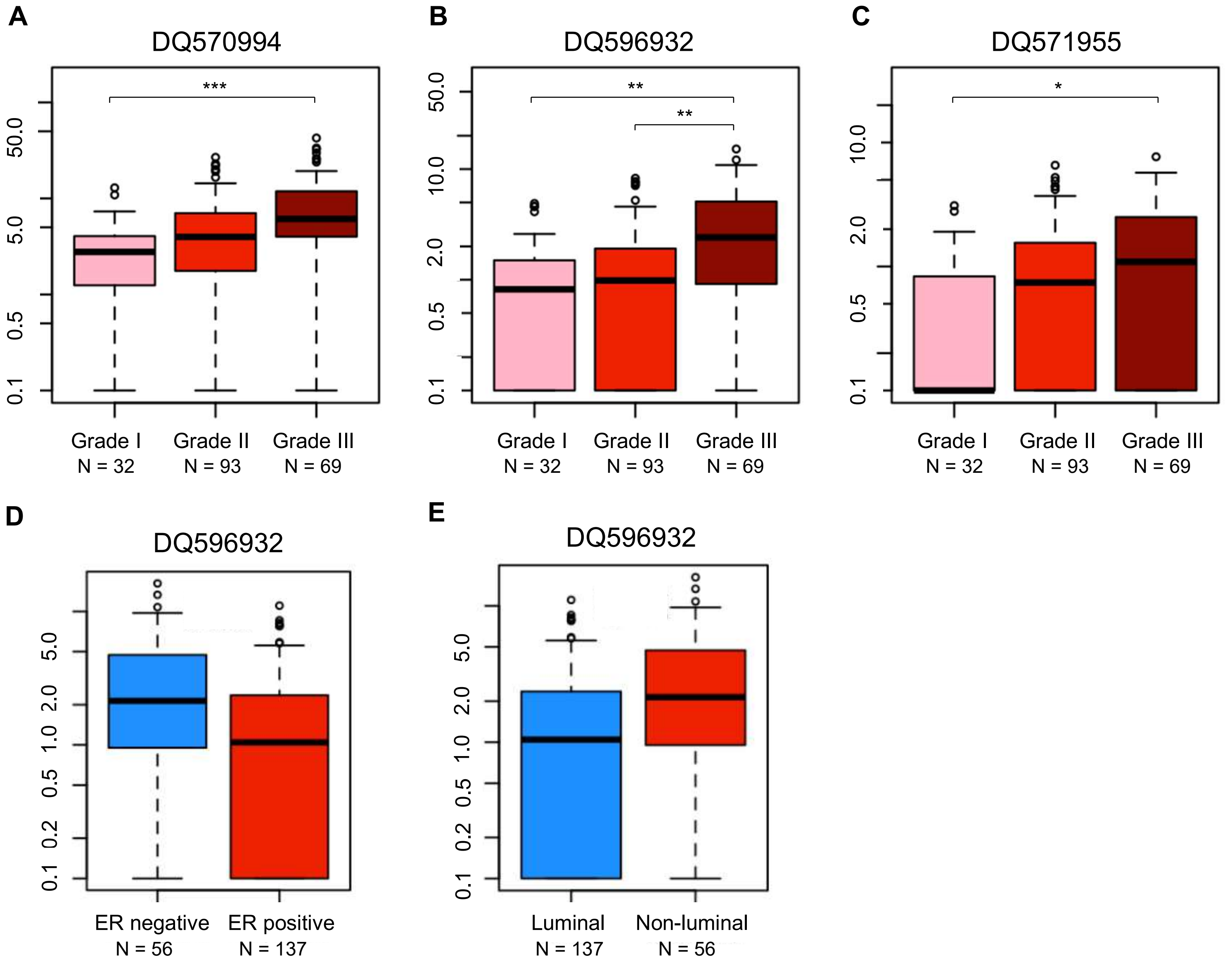

Supplementary Figure S6. The significant associations of the observed RNAs with the clinicopathological features of breast cancer. The upregulation of **(A)** DQ570994 ($Log_2FC=1.33$, *Padj*=0.0001), **(B)** DQ596932 ($Log_2FC=1.37$, *Padj*=0.0018), and **(C)** DQ571955 ($Log_2FC=1.25$, *Padj*=0.023) was observed in the grade III tumors compared to the grade I tumors in all invasive breast cancer cases. Additionally, DQ596932 was upregulated **(B)** in the grade III tumors compared to the grade II tumors ($Log_2FC=1.00$, *Padj*=0.0034), **(D)** in the ER negative tumors compared to the ER positive tumors ($Log_2FC=0.94$, *Padj*=0.036), and **(E)** in non-luminal breast cancer compared to luminal breast cancer ($Log_2FC=0.94$, *Padj*=0.036) in all invasive breast cancer cases.

*** Adjusted *P*<0.001
** Adjusted *P*<0.01
* Adjusted *P*<0.05

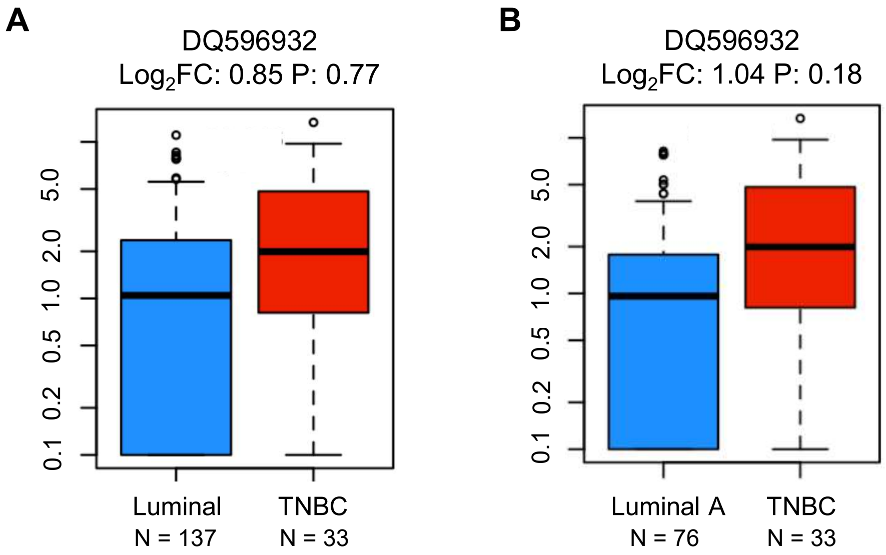

Supplementary Figure S7. The potential association of DQ596932 with TNBC. **(A)** DQ596932 showed a trend towards its upregulation in TNBC (Log_2FC=0.851, *P*=3.86e-03), when compared to luminal breast cancer, although the adjusted *P*-value (0.767) did not reach statistical significance. **(B)** DQ596932 showed a trend towards its upregulation in TNBC (Log_2FC=1.045, *P*=8.85e-04) also when the reference group included only the luminal A cases, although the adjusted *P*-value (0.176) was still not statistically significant.

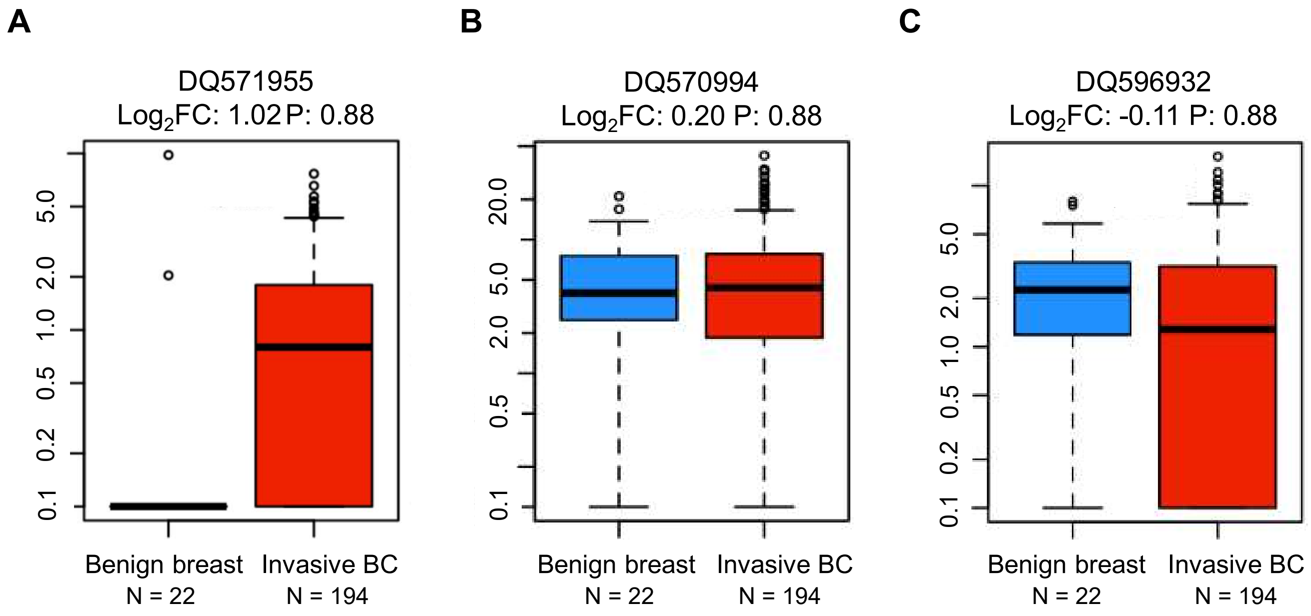

Supplementary Figure S8. The levels of the observed three RNAs in the comparison of invasive breast cancer vs. benign breast tissue. **(A)** DQ571955 seemed to be present exclusively in invasive breast cancer when compared to benign breast tissue, although the *P*-values did not reach statistical significance (*P*=0.248 and *Padj*=0.879). The difference in the presence of **(B)** DQ570994 and **(C)** DQ596932 between invasive breast cancer and benign breast tissue was not as pronounced as for DQ571955.

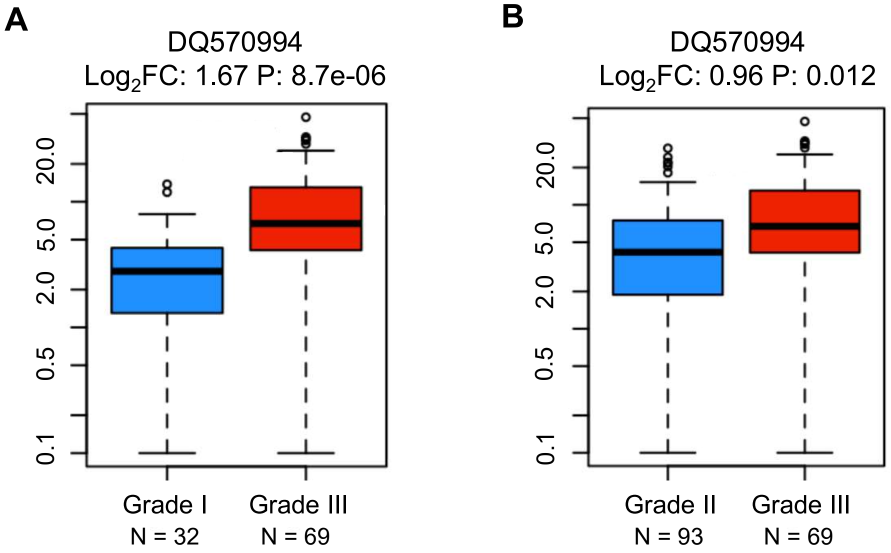

Supplementary Figure S9. The association of DQ570994 with the tumor grade is independent of the ER status. DQ570994 was upregulated in the grade III tumors compared to **(A)** the grade I tumors and **(B)** the grade II tumors in all invasive cases, when the ER status was included as a covariate in the analysis.
