# Supplementary material for: The debatable presence of PIWI‐interacting RNAs in invasive breast cancer
Source: Cancer Med. 2021 May 7;10(11):3593–603. doi: 10.1002/cam4.3915 (PMC8178507; doi:10.1002/cam4.3915)
Supplement: Supplementary file 4 — Fig S10‐15 [file CAM4-10-3593-s005.pdf]

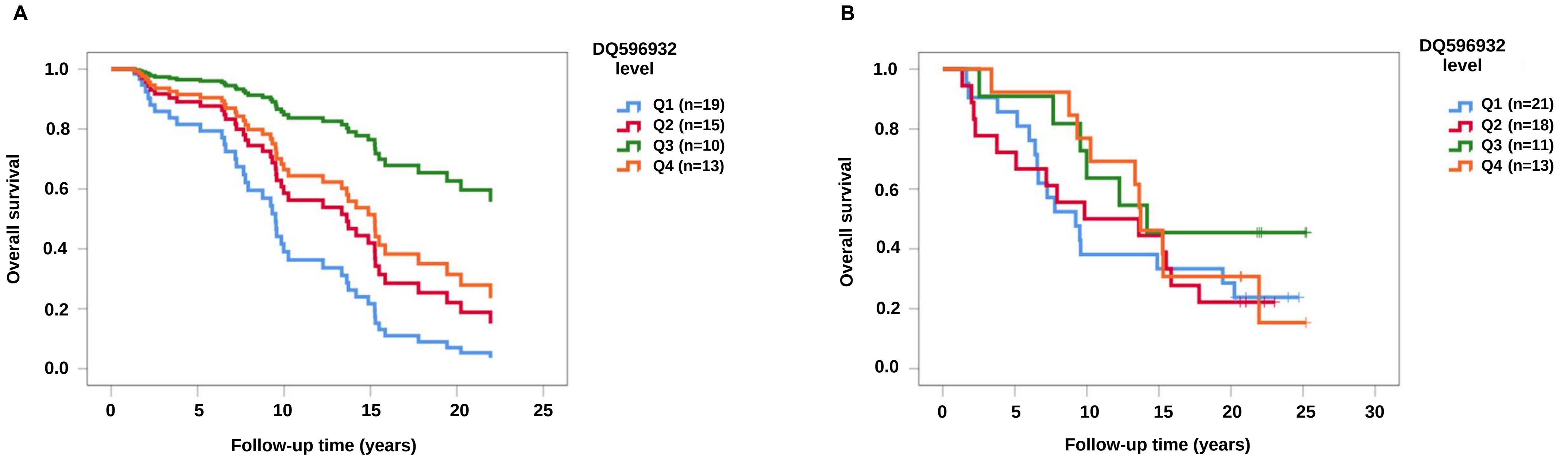

Supplementary Figure S10. The association of DQ596932 with OS in the ER positive RT-treated cases. **(A)** The higher quartile (Q3) of DQ596932 significantly associated with better OS [*P*=0.0067, HR (CI 95%) = 0.20 (0.06-0.64)], when compared to the lowest quartile (Q1) in the ER positive RT-treated cases (n=57) in the Cox multivariate analysis. **(B)** Kaplan-Meier plot showing the association of DQ596932 with OS, which did not reach statistical significance [Overall Log Rank *P*=0.509, for Q3 *P*=0.174, HR (CI 95%) = 0.52 (0.20-1.33)] in the ER positive RT-treated cases (n=63) in the univariate survival analysis.

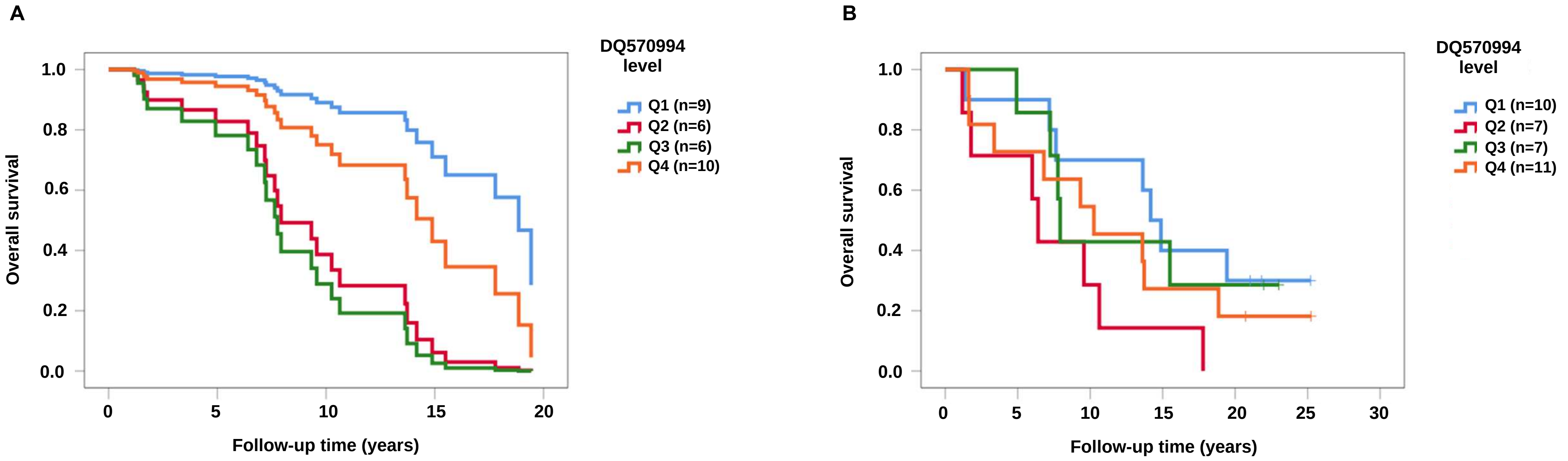

Supplementary Figure S11. The association of DQ570994 with OS in the tamoxifen-treated cases. **(A)** The higher quartiles (Q2 and Q3) of DQ570994 significantly associated with poorer OS [*P*=0.0131, HR (CI 95%) = 7.61 (1.53-37.85) for Q2 and *P*=0.0124, HR (CI 95%) = 8.16 (1.57-42.31) for Q3], when compared to the lowest quartile (Q1) in the tamoxifen-treated cases (n=31) in the Cox multivariate analysis. **(B)** Kaplan-Meier plot showing the significant association of elevated DQ570994 quartile (Q2) with OS [for Q2 *P*=0.0402, HR (CI 95%) = 3.08 (1.05-9.03)] in the tamoxifen-treated cases (n=35). Association of higher quartile (Q3) compared to Q1 and the overall association were not statistically significant [for Q3 *P*=0.733, HR (CI 95%) = 1.22 (0.39- 3.86) and Overall Log Rank *P*=0.174].

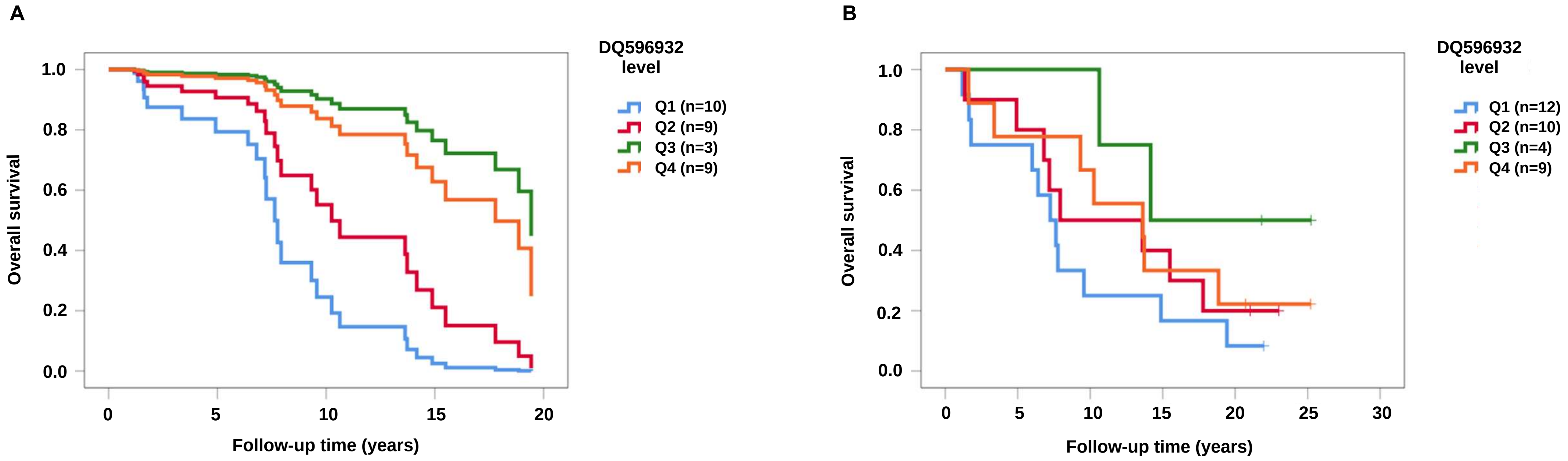

Supplementary Figure S12. The association between DQ596932 and OS in the tamoxifen-treated cases. **(A)** The highest quartiles (Q3 and Q4) of DQ596932 significantly associated with better OS [*P*=0.0422, HR (CI 95%) = 0.11 (0.01-0.93) for Q3 and *P*=0.0063, HR (CI 95%) = 0.16 (0.04-0.60) for Q4], when compared to the lowest quartile (Q1) in the tamoxifen-treated cases (n=31) in the Cox multivariate analysis. **(B)** The highest DQ596932 quartiles (Q3 and Q4) seemed to associate with better OS in the tamoxifen-treated cases (n=35) also in the univariate analysis, even though the association did not reach statistical significance [Overall Log Rank *P*=0.254, for Q3 *P*=0.0785, HR (CI 95%) = 0.26 (0.06-1.17), and for Q4 *P*=0.231, HR (CI 95%) = 0.56 (0.21-1.45)].

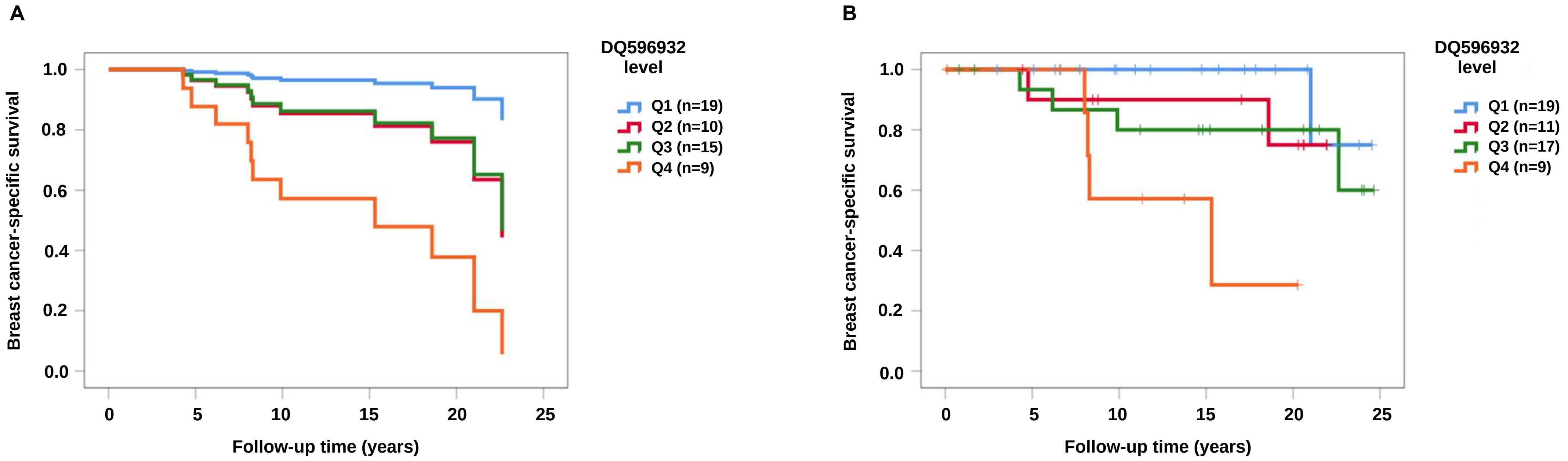

Supplementary Figure S13. The association of DQ596932 with BCSS in the ER positive, surgery-only cases. **(A)** The highest quartile (Q4) of DQ596932 significantly associated with poorer BCSS [*P*=0.0171, HR (CI 95%) 15.65 = (1.63-150.06)], when compared to the lowest quartile (Q1) in the ER positive, surgery-only cases (n=53) in the Cox multivariate analysis. **(B)** Kaplan-Meier plot showing the significant association between the highest quartile of DQ596932 and poorer BCSS [Overall Log Rank *P*=0.025, for Q4 *P*=0.0188, HR (CI 95%) = 14.75 (1.56-139.35)] in the ER positive, surgery-only cases (n=56) in the univariate analysis.

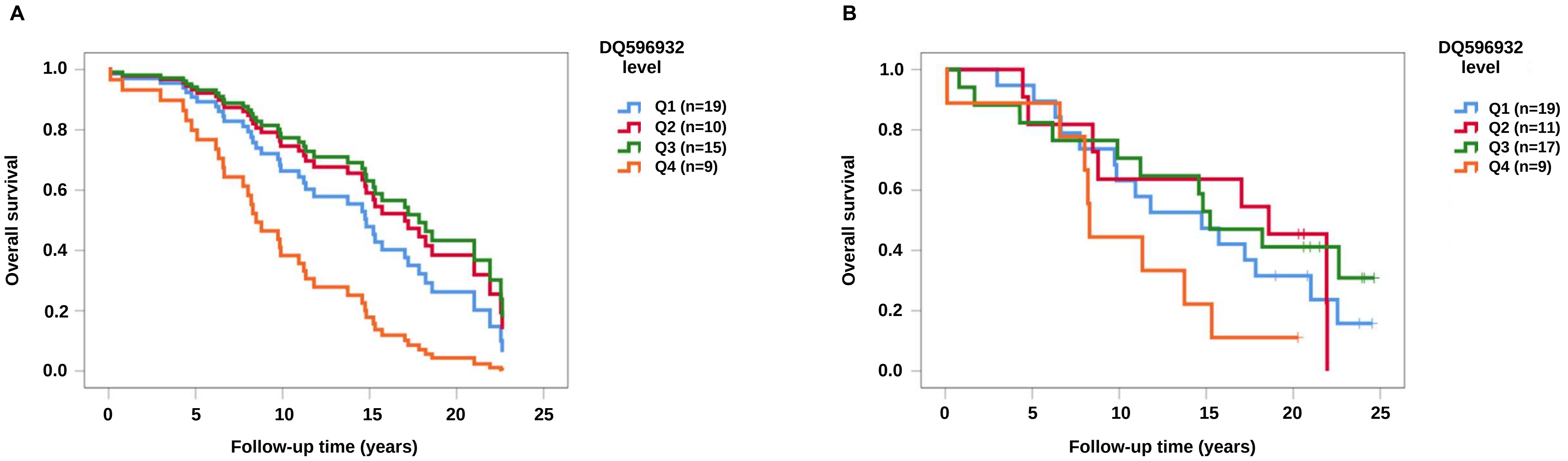

Supplementary Figure S14. The association between DQ596932 and OS in the ER positive, surgery-only cases. **(A)** The highest quartile (Q4) of DQ596932 associated with poorer OS [*P*=0.0343, HR (CI 95%) = 3.00 (1.08-8.28)], when compared to the lowest quartile (Q1) in the ER positive, surgery-only cases (n=53) in the Cox multivariate analysis. **(B)** The highest quartile (Q4) of DQ596932 seemed to associate with poorer OS in the ER positive surgery only cases (n=56) also in the univariate survival analysis, even though the association did not reach statistical significance (Overall Log Rank *P*=0.203, for Q4 *P*=0.146, HR (CI 95%) = 1.92 (0.80-4.62)].

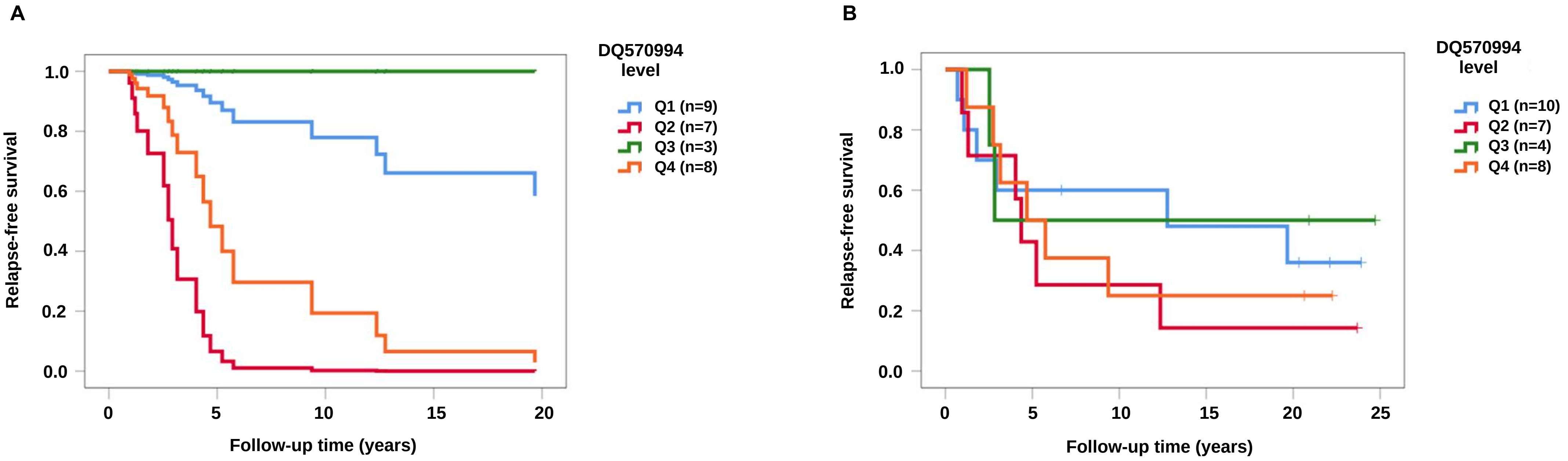

Supplementary Figure S15. The association of DQ570994 with RFS in the CT-treated cases. **(A)** The higher quartile (Q2) of DQ570994 significantly associated with poorer RFS [*P*=0.0281, HR (CI 95%) = 5.36 (1.20-24.03)], when compared to the lowest quartile (Q1) in the adjuvant CT-treated cases (n=27) in the Cox multivariate analysis. **(B)** In the univariate analysis, the association of DQ570994 with RFS in the adjuvant CT-treated cases (n=29) did not reach statistical significance [Overall Log Rank *P*=0.708, for Q2 *P*=0.331, HR (CI 95%) = 1.76 (0.56-5.54)].
